# Comparison of Two Dosing Regimens of Miltefosine, Both in Combination With Allopurinol, on Clinical and Parasitological Findings of Dogs With Leishmaniosis: A Pilot Study

**DOI:** 10.3389/fvets.2020.577395

**Published:** 2020-12-14

**Authors:** Fabrizio Iarussi, Paola Paradies, Valentina Foglia Manzillo, Manuela Gizzarelli, Mariano Francesco Caratozzolo, Christelle Navarro, Beatrice Greco, Giuseppe Tommaso Roberto Rubino, Gaetano Oliva, Mariateresa Sasanelli

**Affiliations:** ^1^Dipartimento Dell'Emergenza e dei Trapianti di Organi, Sezione Veterinaria, Università Degli Studi “Aldo Moro”, Bari, Italy; ^2^Dipartimento di Medicina Veterinaria e Produzioni Animali, Università Degli Studi di Napoli Federico II, Naples, Italy; ^3^Istituto di Biomembrane, Bioenergetica e Biotecnologie Molecolari, Consiglio Nazionale Delle Ricerche, Bari, Italy; ^4^Virbac SA Global Medical Department, Carros, France

**Keywords:** canine leishmaniosis, modified miltefosine dosage, treatment efficacy, digestive tolerance, combination therapy

## Abstract

Miltefosine (MIL)–allopurinol combination therapy administered at standard dosage is effective to treat canine leishmaniosis, nevertheless for some dogs the digestive tolerance of MIL is not acceptable. This study evaluates an alternative therapeutic protocol by using a modified dosage of MIL to increase its effectiveness and improve the digestive tolerance. Thirty-four *Leishmania infantum* owned naturally infected dogs were included and monitored for 180 days. The dogs were allocated in two randomized groups: Group X−18 dogs treated with MIL registered dose of 2 mg/kg, oral administration, once daily, for 28 days; Group Y−16 dogs treated with 1.2 mg/kg for 5 days followed by 2.5 mg/kg for 25 days. Both groups were also treated with allopurinol. Digestive tolerance was monitored by adverse events observation. Treatments effectiveness was evaluated by monitoring the reduction of clinical score, the improvement of clinicopathological abnormalities, the reduction of parasitological load by PCR and the number of relapses. 16.6% dogs of group X and 12.5% dogs of group Y showed treatment associated adverse events. The reduction of clinical score was 61.7% for group X and 71.6% for group Y. All dogs showed an improvement of laboratory parameters after treatment. Quantitative PCR showed better results in group Y compared to group X; relapses were only registered in four dogs of group X. The modified protocol demonstrates a better trend of results in term of tolerance, clinical effectiveness, parasitological load reduction and relapses control, suggesting it could be considered for new large-scale studies.

## Introduction

Canine leishmaniosis (CanL), caused by the protozoan parasite *Leishmania infantum*, is endemic in southern Italy (1). Dogs that are exposed to the parasite infection could develop clinical signs, remain asymptomatic carriers or even clear the infection due to the individual effective immuno-response (2–4).

Different drugs and protocols have been proposed for CanL treatment (5). Following treatment parasitological healing is possible, but not frequent (6–8); after a temporary remission of clinical signs, disease recurrence can be seen (6, 9–13). N-methylglucamine antimoniate (MA) and miltefosine (MIL) both combined with allopurinol are first-line drug therapies (6, 14–17). Despite its proven efficacy, MA has some drawbacks, including the high costs, the parenteral administration and the numerous reported side effects (18). MIL used at the registered dosage of 2 mg/Kg/die in association with allopurinol, is a good alternative (19–22). Indeed, unlike MA, this molecule has a low impact on renal function and can be used in dogs with proteinuria, one of the most frequent clinicopathological alteration reported in CanL (23, 24). MIL is administered orally, and this is widely preferred by owners. However, adverse reactions associated with MIL treatment have been reported in 11.7% (11/94) to 100% (17/17) of treated dogs (19, 25). Dogs may show gastrointestinal reactions such as vomiting, diarrhea, abdominal pain and loss of appetite that tend to regress spontaneously within a few days after starting treatment, probably because dogs become habituated to the medication (19, 25, 26). MIL adverse events are dose-dependent; at a dosage of 4 mg/kg/die they are observed more frequently than at registered dosage, while they are absent at 1 mg/kg/die, representing the NOEAL dosage for MIL (27). There are also suggestions that a 25% dose rate higher than currently recommended, may be more effective [(15), personal communication]. The authors hypothesize that a particular therapeutic scheme that considers an initial lower dosage followed by an higher one, may allow a better drug tolerance in the reduction of side effects and a better efficacy on parasite load. The aim of this pilot study is to compare the tolerance and efficacy of two different dose rates of MIL (Milteforan®, Virbac, France) in dogs with CanL exhibiting moderate to severe clinical signs of the disease.

## Materials and Methods

### Inclusion/Exclusion Criteria

CanL positive dogs of different breeds and sex, between 1 and 8 years were included in the study. Their owners were asked to sign an informed consent in which all the clinical procedures and the timing of clinical examination were reported. Dogs were included in the trial if they exhibited CanL classified as stages II or III (IRIS I and II) according to the LeishVet classification (15). All dogs resulted positive to direct observation of *Leishmania* spp. amastigotes on fine needle aspirate lymph node smears (28). Dogs were serologically positive at *Leishmania infantum* immunofluorescence antibody test (IFAT–cut off: 1:80), and serologically negative for other vector-borne diseases (VBDs: Anaplasmosis, Ehrlichiosis, Lyme disease, Dirofilariasis) (4Dx® Plus, Idexx). Dogs presenting the following characteristics were not included in the trial: females known to be pregnant or lactating; dogs treated with drugs of known efficacy against CanL within 3 months prior to inclusion; dogs treated with systemic long-acting corticosteroids and other immunomodulatory drugs within 1 month prior to inclusion; dogs with concomitant disorders that may interfere with the evaluation of response to treatment; dogs with life-threatening diseases.

Dogs presenting the following characteristics after treatment administration were excluded during the follow-up: concomitant disorders that may interfere with the evaluation of response to treatment, adverse events that required stopping the treatment or the follow-up, failure of compliance to the protocol.

### Groups and Treatment

Dogs were fairly divided in two treatment groups (see below) following a tiered randomization system, by using the table of randomization. Dogs were housed, managed and fed regularly at owners' facilities. All dogs received a permethrin-based spot-on (Exspot®, Schering Plow Animal Health) during the whole study period, to reduce the risk of other VBDs.

The two groups were treated as follows:

Group X: Miltefosine: 2 mg/kg BW, orally, once a day, for 28 consecutive days (Milteforan®, Virbac, France).

Group Y: Miltefosine: 1.2 mg/kg BW, orally, once a day, for 5 consecutive days then 2.5 mg/kg BW, orally, once a day, for 25 consecutive days (Milteforan®, Virbac, France).

In addition, all dogs received allopurinol (Zyloric®, Teofarma, Italy) at a dose rate of 10 mg/kg BID (14, 15) for 180 days.

### Clinical and Laboratory Monitoring

The dogs were observed for 180 days (D). At D0, D30, D60, D90, and D180 clinical scores and body weight were registered on the individual files. Clinical score was obtained by evaluating the presence of 26 clinical manifestations according to Mirò et al. (18). The severity of each sign was assessed with a score from 0 (absence of clinical sign) to 3 (severe clinical sign). At the same time points blood samples were collected for complete blood count (CBC), clinicopathological findings [e.g., urea, creatinine, total proteins, albumin concentration, globulin concentration and fractions, albumin/globulin ratio, protein electrophoresis, alanine-aminotransferase (ALT), alkaline-phosphatase (ALP), total bilirubin] and IFAT for *Leishmania infantum*. At D0 and D60 bone marrow (BM) samples were collected for qPCR analysis from the sternum bones, following the procedure described by Paparcone et al. (29). At D0, D30, and D60, urine samples were collected by ultrasound-guided cystocentesis for a complete urinalysis and urinary protein creatinine (UPC) ratio. At D60 the popliteal lymph-nodes were sampled by fine needle aspiration for microscopic detection of *Leishmania* amastigotes (30).

### Criteria to Assess Treatment Efficacy and Tolerance

#### Clinical Score and Laboratory Findings

For each dog, the percentage of reduction of the clinical score at D180, when compared to D0, was calculated. Furthermore, the improvement and/or the normalization of laboratory parameters were investigated.

#### Serological and Parasitological Diagnosis

Detection of anti-*Leishmania* IgG antibodies was performed by an in-house IFAT assay using *Leishmania infantum* promastigotes (WHO reference strain MHOM/TN/1980/IPT-1) as antigen and following the protocol recommended by the Office International des Epizooties (31) The cut-off dilution was set at 1:80. BM aspirate material was examined by q-PCR assay following the methodology used by the Italian National Reference Center for Leishmaniosis (C.Re.Na.L, Palermo, Italy) (32). Briefly, *Leishmania* DNA derived from the patient's bone marrow was subjected to two consecutive PCR amplifications using the kinetoplastid-specific primers R221 and R332 in the first run, and the *Leishmania*-specific primers R223 and R333 in the second run (33).

#### Relapses

Clinical, laboratory and serological parameters were analyzed at each follow up to identify dogs under relapse. In case of suspect, a further 4Dx test was performed to exclude other or concomitant VBDs. The number and the timing of relapses for each treatment group were evaluated and compared at the end of the study. The recurrence of clinical signs and clinicopathological alterations suggestive of leishmaniosis was considered as relapse. Clinical and clinicopathological modifications were chosen as the main findings suggesting relapses, together with a positive lymph node cytology. These findings were not always associated to the elevation of the antibody titers, the increasing of which is considered a not constant marker of relapse (34, 35).

#### Treatments Safety and Tolerance

The safety-tolerance of both treatments was assessed by the incidence of adverse events observed daily from the owner during the treatment: percentage, duration, severity, relationship to the treatment. The assessment of urea, creatinine and hepatic enzymes as well as UPC ratio before and after treatment, were also considered to investigate the safety of the two protocols.

### Statistical Analysis

Statistical analysis of clinical and laboratory results was performed with NCSS and SAS software. For each parameter, which follow a normal distribution, the two groups were compared at baseline using a Student's *t*-test. Mixed model analysis of variance was used to compare the two groups over time. To complete the analysis of score evolution, between the two groups, Wilcoxon's test was used to compare score change between D0 and D180, for non-normal distribution data.

Furthermore, the incidence of adverse effects in the two groups of treatment were compared by using a Student's *t*-test (*p*-value < 0.05).

The Real Time PCR data are represented in box-and-whisker plots showing median and 10, 25, 75, and 90th percentiles for each sample category. Number of particles/ml were considered as absolute numbers for each sample; two-tailed Student's *t*-tests were performed to assess the statistical significance of the differences observed. The level of significance was set at a *p*-value < 0.05.

## Results

A total of 34 dogs met the inclusion criteria and were enrolled in the study. They were randomly distributed in the two treatment groups, as follows: 18 dogs in group X (10 dogs in Leishvet Stage II and eight dogs in Leishvet Stage III) and 16 dogs in group Y (eight dogs in Leishvet Stage II and eight dogs in Leishvet Stage III). Dogs in group X assumed a total dosage of MIL of 56 mg/kg (100%) while dogs in group Y assumed atotal dosage of 68.5 mg/kg (122%). Dog data, clinical score, laboratory parameters and Leishvet classification for both groups at inclusion are reported in Tables 1A,B, respectively. Thirty-one out of the 34 enrolled dogs (91.2%) completed the trial. Three dogs from group X did not reach D180; one dog (16X) showed signs of relapse at D90 and two dogs died between D90 and D180 (7X−9X) for reasons not related to CanL.

**Table 1:** **(A,B):** Inclusion criteria of dogs treated by standard (group X) or modified (group Y) dosage of miltefosine.

**Table 1A**												
**Case code**	**Breed**	**SexAge**	**Weight (kg)**	**Clinical score**	**HCT (37–55%)**	**Pt (6.0–8.0 gr/dl)**	**Alb (3.06–4.72 gr/dl)**	**A/G (0.50-1.30)**	**Crea (1.0–2.0 mg/dl)**	**UPC < 0.5**	**Reciprocal IFAT titer**	**Leishvet clinical stage**
1X	Cross	M/n6y	18	4	55.9	6.4	2.85	0.8	1.1	0.15	320	II
2X	E. Setter	M4y	18	6	52.7	6.8	3.37	0.98	0.98	0.6	160	II
3X	Maremma S.	F8y	32	22	51.5	9.2	2.67	0.41	1.15	1.67	320	III IRIS 1
4X	Great Dane	M4y	50	8	29	11.7	1.85	0.19	1.01	2.25	320	III IRIS 1
6X	Breton	M5y	17	5	29.2	7.14	2.26	0.46	0.6	1.42	320	III IRIS 1
7X	Cross	F5y	22.5	7	41.3	7.51	2.07	0.38	0.93	0.3	320	II
8X	Cross	M6y	23	13	30	8	1.23	0.18	0.81	3.97	320	III IRIS 1
9X	Cross	M/n3y	15	7	33.9	7.5	2.24	0.42	1.68	3.64	320	III IRIS 2
10X	Doberman P.	F7y	18	13	25.4	10.8	2.29	0.27	1.02	1.23	320	III IRIS 1
11X	B. Collie	F6y	10	10	26.1	7.1	2.00	0.39	0.59	5.34	320	III IRIS 1
12X	E. Setter	M4y	16	4	43.8	7.9	0.00	0.44		0.22	320	II
13X	Dogue de B.	M3y	38	11	39.9	9.4	1.44	0.18	1.78	4.2	320	III IRIS 2
14X	Cross	F6y	20	8	35.8	7.4	2.35	0.47	1.12	0.52	320	II
15X	Cross	M/n7y	25	3	35.7	7.4	2.47	0.5	0.85	0.2	320	II
16X	German S.	M/n5y	22	6	33.5	6.8	2.16	0.46	1.23	0.58	160	II
17X	Cross	F4y	16.8	7	37.6	7.4	1.73	0.31	1.08	0.31	160	II
18X	Cross	F3y	32	6	40.3	7.5	2.48	0.49	0.81	0.51	160	II
19X	Cross	M/n5y	24	4	33.7	7.8	2.32	0.42	0.99	0.28	320	II
**Table 1B**												
**Case code**	**Breed**	**SexAge**	**Weight (kg)**	**Clinical score**	**HCT (37–55%)**	**Pt (6.0–8.0 gr/dl)**	**Alb (3.06–4.72 gr/dl)**	**A/G (0.50–1.30)**	**Crea (1.0–2.0 mg/dl)**	**UPC** **<** **0.5**	**Reciprocal IFAT titer**	**Leishvet clinical stage**
1Y	Cross	M3y	17.5	8	29.7	11.7	2.06	0.21	1.02	1.96	320	IIIIRIS 1
2Y	Cross	F5y	14.5	11	32	9	1.92	0.27	0.81	1.58	320	IIIIRIS 1
3Y	Cross	M5y	21.5	13	37.2	11	2.22	0.25	0.86	0.69	640	II
4Y	Cross	M4y	22	7	30.2	11	2.07	0.23	1.24	0.55	160	II
5Y	German S.	M7y	28	7	45.7	7.6	2.59	0.52	1.05	0.23	320	II
6Y	Cross	F9y	21	13	36.6	9.2	2.32	0.34	0.81	0.63	320	II
7Y	G. Dane	F2y	38	21	24.1	12.2	1.6	0.15	0.94	3.37	320	II
8Y	Cross	M3y	4	10	20.1	7.8	1.48	0.23	1.72	25	320	IIIIRIS 2
9Y	Setter	M5y	15	19	27.2	8	1.13	0.16	0.41	1.46	320	IIIIRIS 1
10Y	B. Collie	F4y	21	9	36	7.4	2.31	0.45	0.83	0.15	320	II
11Y	J. Russel	M5y	8.3	8	44.7	7	2.3	0.53	0.87	0.39	320	II
12Y	Labrador	M3y	31	2	43.8	5.8	2.34	0.49	1.07	0.18	320	II
13Y	Cross	M5y	16	7	30	9.2	1.82	0.25	0.75	3.3	320	IIIIRIS1
14Y	Cross	M6y	14	5	32.8	8.8	1.69	0.24	2	1.9	320	IIIIRIS2
15Y	Cross	F3y	26.5	9	37.4	7.2	2.10	0.41	0.96	1.55	320	IIIIRIS1
16Y	Pincher	F5y	3	13	28.4	8.4	1.74	0.26	0.73	183	320	IIIIRIS1

*Dogs were clinically classified according to Leishvet and IRIS stages*.

### Clinical Score

**Figure 1:**
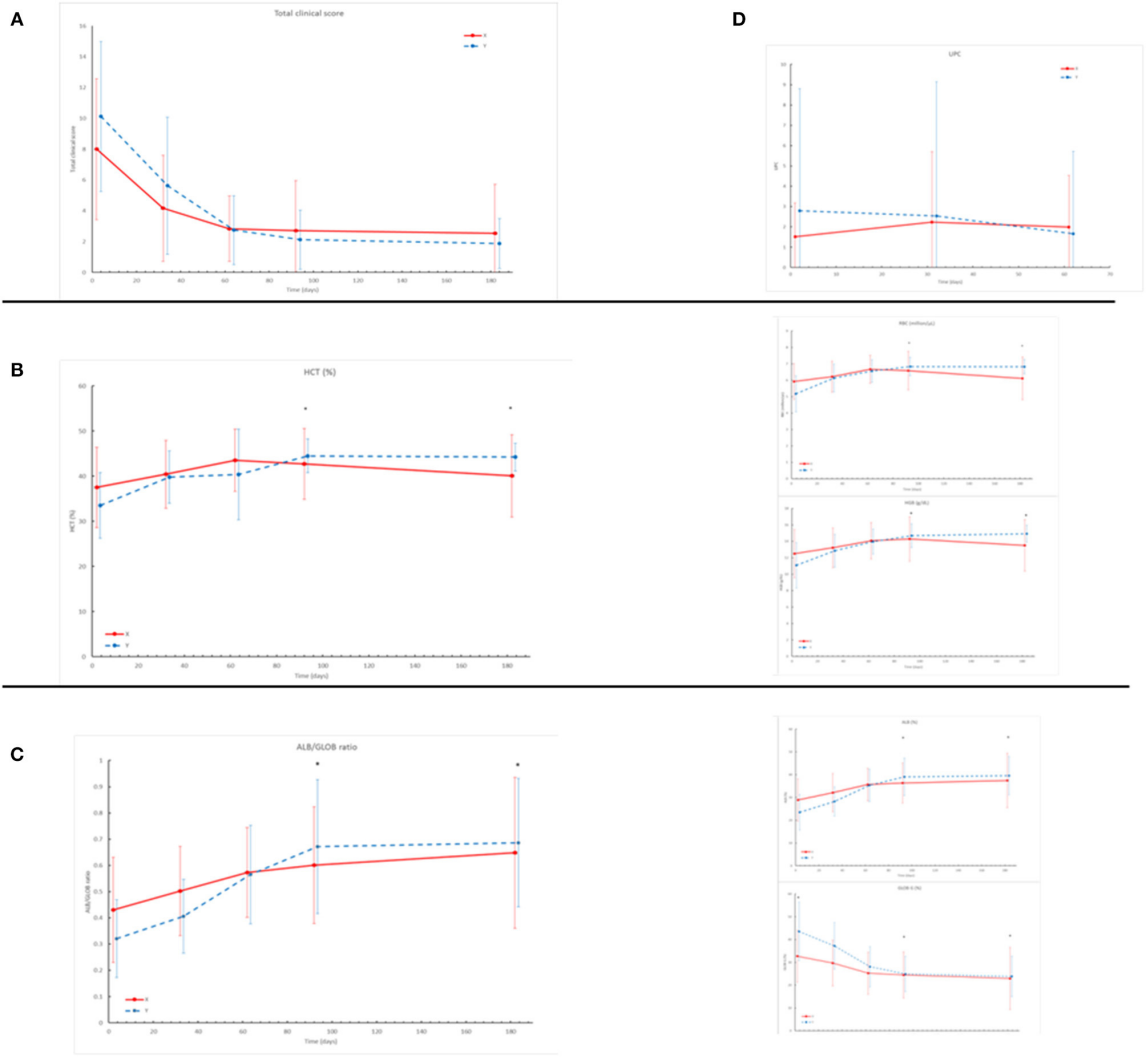
**(A)** The Clinical Score shows severe reduction throughout the study in both groups (X red line, Y blue line). No statistical differences were registered between the two groups **(B)** HCT%, Hb (g/dl), RBC (× 10^6^/ml) registered in the two groups throughout the study. The asterisk shows statistical differences (D90, D180): HCT%: *P* = 0.0005 RBC: *P* < 0.0001 Hgb: *P* = 0.0001. **(C)** Albumin/globulin ratio, Albumin%, Globulin% registered in the two groups throughout the study. The asterisk shows statistical differences (D90/D180): Alb/Glob: *P* = 0.01; Alb%: *P* = 0.004; Glob%: *P* = 0.003. **(D)** Results of UPC at urinalysis showed a trend of reduction in group Y and a trend of increase in group X throughout the study (no statistical differences registered).

The clinical score reduced within 2 months after treatment (Figure 1A). The percentage of clinical score reduction from D0 to D180 was 61.7% in group X and 71.6% in group Y. The statistical analysis of the total clinical score and the percentage of its reduction did not show significant differences between the two groups.

### Clinicopathological Findings

Mean values of clinical pathological parameters at each follow up in the two groups are reported in Table 2. Statistically significant differences were identified between the two groups on D90 and D180 for the values of Hematocrit (HCT%), Red Blood Cell (RBC), Hemoglobin (Hgb) (Figure 1B), Albumin (Alb%), Globulin (Glob%), and A/G ratio (Figure 1C). Differently, there was no significant difference (*p* > 0.05) regarding renal and hepatic parameters during the study. However, UPC ratio results showed a trend of reduction in group Y and increase in group X, despite no significant differences were detected (Figure 1D).

**Table 2:** Mean values and standard deviation of clinicopathological parameters in the two groups (X, Y) at different times.

**Time/group**	**Hct%**	**Rbc (M/ μ L)**	**Hgb (g/dl)**	**Plt (k/ μ L)**	**Urea (mg/dl)**	**Crea (mg/dl)**	**Alt (IU/L)**	**Bil. Tot (mg/dl)**	**TP (g/dl)**	**Alb%**	**Alb (mg/dl)**	**Glob (%)**	**A/G ratio**	**IFAT titer**	**UPC**
D0X	37.5 ± 8.93	5.9 ± 1.09	12.4 ± 2.92	242.7 ± 120.88	40.9 ± 22.02	1.04 ± 0.31	37.7 ± 22.56	0.06 ± 0.03	8.0 ± 1.40	28.8 ± 9.23	2.09 ± 0.71	32.7 ± 11.42	0.43 ± 0.19	284.4 ± 68.44	1.52 ± 1.65
D30X	40.4 ± 7.5	6.2 ± 0.94	13.2 ± 2.41	201.6 ± 100.87	39.2 ± 21.60	1.09 ± 0.29	33.2 ± 15.87	0.07 ± 0.04	7.8 ± 1.29	32.1 ± 8.40	2.45 ± 0.55	29.7 ± 10.02	0.50 ± 0.17	306.6 ± 56.56	2.23 ± 3.45
D60X	43.5 ± 6.9	6.6 ± 0.84	14.0 ± 2.20	199.6 ± 91.50	43.5 ± 32.01	1.14 ± 0.33	51.7 ± 61.12	0.06 ± 0.05	7.6 ± 0.95	35.7 ± 7.13	2.71 ± 0.55	25.2 ± 9.16	0.057 ± 0.17	297.7 ± 66.11	1.98 ± 2.53
D90X	42.7 ± 7.8	6.5 ± 1.17	14.28 ± 2.69	206.8 ± 96.44	36.7 ± 16.51	1.09 ± 0.22	51.2 ± 48.89	0.09 ± 0.06	7.2 ± 0.99	36.3 ± 8.81	2.62 ± 0.60	24.4 ± 10.11	0.60 ± 0.22	300.0 ± 54.65	−
D180X	40.1 ± 9.1	6.1 ± 1.29	13.5 ± 3.12	213.73 ± 134.38	40.0 ± 17.64	1.04 ± 0.29	42.0 ± 24.22	0.06 ± 0.04	7.3 ± 1.74	37.4 ± 11.94	2.63 ± 0.64	22.9 ± 13.65	0.64 ± 0.28	268.5 ± 86.54	−
D0Y	33.5 ± 7.29	5.1 ± 1.09	11.0 ± 2.75	324.8 ± 255.30	44.43 ± 41.5	1.00 ± 0.38	36.6 ± 18.60	0.06 ± 0.02	8.8 ± 1.82	23.5 ± 7.80	1.97 ± 0.38	43.5 ± 12.85	0.32 ± 0.14	330.0 ± 91.79	2.79 ± 6.00
D30Y	39.8 ± 5.81	6.1 ± 0.84	12.8 ± 1.98	338.1 ± 152.27	38.0 ± 19.35	1.00 ± 0.24	56.1 ± 40.85	0.05 ± 0.03	8.6 ± 1.42	28.2 ± 6.41	2.37 ± 0.32	37.2 ± 10.23	0.40 ± 0.14	310.0 ± 40.00	2.53 ± 6.60
D60Y	40.4 ± 10.03	6.5 ± 0.68	13.9 ± 1.51	316.5 ± 111.79	40.5 ± 15.06	1.06 ± 0.21	39.4 ± 15.82	0.05 ± 0.02	7.5 ± 0.96	35.3 ± 7.13	2.63 ± 0.46	28.0 ± 8.84	0.56 ± 0.18	300.0 ± 54.65	1.66 ± 4.06
D90Y	44.5 ± 3.74	6.8 ± 0.56	14.6 ± 1.44	302.1 ± 114.01	42.5 ± 18.78	1.01 ± 0.17	60.6 ± 34.64	0.06 ± 0.05	7.2 ± 0.80	39.0 ± 8.23	2.80 ± 0.52	24.8 ± 7.74	0.67 ± 0.25	290.0 ± 64.49	−
D180Y	44.2 ± 3.06	6.8 ± 0.43	14.9 ± 1.03	304.6 ± 140.79	35.2 ± 7.38	0.99 ± 0.15	46.4 ± 26.35	0.07 ± 0.03	7.3 ± 0.99	39.5 ± 8.38	2.86 ± 0.57	23.8 ± 8.86	0.68 ± 0.24	230.0 ± 81.97	−

### IFAT

In all dogs, except for one, IFAT titers were reduced or remained stable till the end of the study. IFAT titers did not show any significant difference between the two groups at D0 and throughout the study.

### Parasitological Results

At D60, all dogs, except for two (1 in group X and 1 in group Y), resulted negative at lymph-node smears examination. Real Time-PCR was performed on a total of 66 BM samples due to an unsuitable material obtained from two dogs of group X at D60.

Real Time-PCR results showed that two out of 14 dogs (14.3%) reached a negative result at D60 in group X vs. seven out of 14 (50%) in group Y (Table 3).

**Table 3:** Leishmania DNA load in Group X and Group Y.

**qPCR**	**GROUP X**		**GROUP Y**	
**Dogs**	**D0**	**D60**	**D0**	**D60**
1	6.6E + 01	6.3E + 02	1.8E + 06	0
2			4.5E + 03	1.8E + 03
3	1.1E + 03	0	4.0E + 06	8.0E + 04
4	1.1E + 07	1.0E + 07	2.6E + 04	0
5			6.6E + 05	4.4E + 04
6	7.6E + 04	1.5E + 05	7.6E + 02	0
7			8.2E + 06	4.1E + 06
8			2.0E + 04	2.3E + 03
9	1.1 + 06	5.1E + 05	3.1E + 06	7.0E + 03
10	6.4E + 06	5.2E + 01	1.4E + 03	0
11	1.6E + 06	9.0E + 04		
12	1.6E + 06	2.3E + 04	3.2E + 05	0
13	5.8E + 06	5.4E + 03	6.1E + 06	0
14	2.5E + 05	3.4E + 04	2.3E + 03	4.9E + 03
15	2.9E + 03	0	3.3E + 02	0
16	1.2E + 05	2.8E + 03		
17	7.0E + 05	2.6E + 03		
18				
19	4.3E + 06	1.8E + 06		

*The parasite load in the bone marrow pre- and post-therapy was measured by RTQ-PCR. Data are reported as number of particles/ml*.

**Figure 2:**
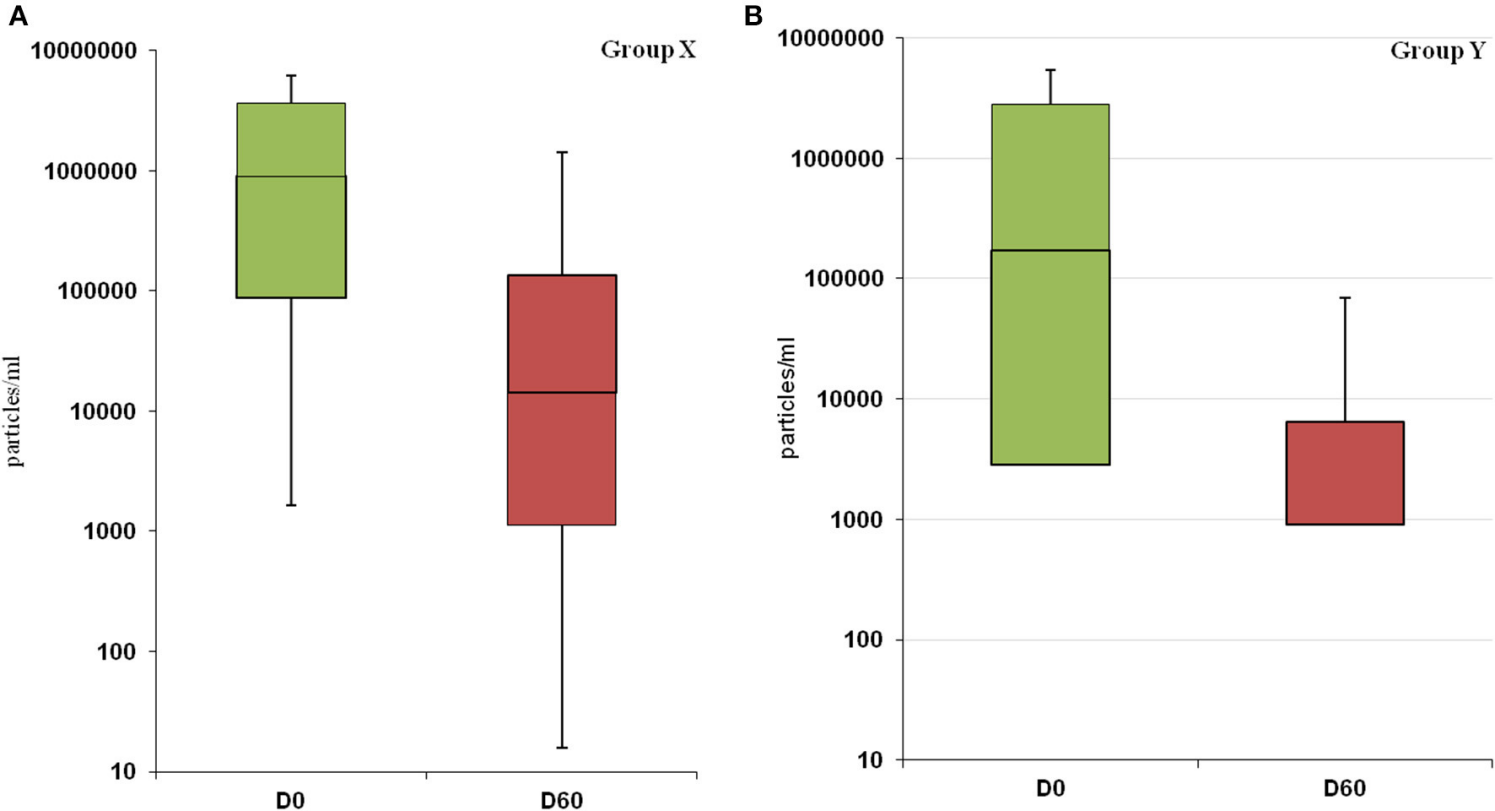
Changes in parasitic load following treatment in group X **(A)** and Y **(B)**. The decrease in parasitic load is not statistically significant in both groups (X: *p*-value = 0.21; Y: *p*-value = 0.08).

Statistical analysis showed that at D0 there was no significant difference between the two groups (*p*-value = 0.58), despite a marked heterogeneity of the parasitic load for both groups. In group X the lowest value was 6.6E + 01 parasitic particles per ml of sample (dog 1X) while the highest value was 1.1E + 07 (dog 4X). In group Y the lowest recorded value was 3.3E + 02 (dog 15Y), the highest 8.2E + 06 (dog 7Y). After treatments, the parasitic load decreased in both groups: group X, from 2.35E + 06 (D0) to 9.01E + 05 (D60); group Y from 1.73E + 06 (D0) to 3.03E + 05 (D60) (Figures 2A,B). We performed a *post-hoc* analysis exclusively on the subjects that presented the parasitic load higher than 10E + 04 particles per ml (10 dogs in group X and seven in group Y). The parasitic load was decreased from an average value at D0 of 2.43E + 06 to a value of 2.74E + 05 at D60 in subgroup X, while in subgroup Y the decrease ranged from 3.21E + 06 at D0 to 6.04E + 05 at D60.

### Relapses

Four relapses were registered throughout the study, all in group X. The owners referred worsening of the clinical picture. During clinical examination weight loss and reduction of HCT% was registered in all dogs compared to previous follow up. Laboratory parameters of these dogs are reported in Table 4. One dog (16X) experienced relapses at D90, the other three dogs (4X, 14X, and 19X) at D 180. In all these four cases lymph node cytology showed the presence of *Leishmania* spp. amastigotes.

**Table 4:** Clinicopathological parameters (bold values) of dogs showing relapses during the study.

**Case code**	**TIME**	**Weight(kg)**	**Clinical Score**	**HCT%**	**TP**	**Alb (mg/dl)**	**γ Glob (%)**	**A/Gratio**	**Urea**	**Crea**	**UPC**	**Reciprocal IFAT titer**
4X	D0	50	8	29	11.7	1.85	58.5	0.19	31	1.01	2.25	320
	D30	51	3	38.5	11.8	1.65	54.1	0.3	36	1.13	1.88	320
	D60	52	4	41.2	10.5	2.98	44.8	0.4	41	1.16	0.91	320
	D90	54	1	41	10	2.57	50.5	0.35	33	1.12	nd	320
	**D180**	**38**	**3**	**34.3**	**13**	**1.90**	**63.7**	**0.17**	**30**	**0.99**	**nd**	**320**
14X	D0	20	8	35.8	7.4	2.35	32.6	0.47	42	1.12	0.52	320
	D30	20	4	41	8.2	3.28	28.6	0.67	49	1.13	0.33	320
	D60	20	3	38.8	8.4	3.18	22.7	0.61	44	1.14	0.47	320
	D90	20	2	38.8	8.4	3.18	22.7	0.61	44	1.14	nd	320
	**D180**	**17**	**12**	**32.9**	**5.6**	**2.68**	**19.3**	**0.92**	**70**	**0.84**	**nd**	**320**
16X	D0	22	6	33.5	6.8	2.16	30.6	0.46	62	1.23	0.58	160
	D30	22	3	37.1	6.6	2.10	30.5	0.47	60	1.54	0.76	320
	D60	22	1	41.3	6.9	2.33	27.2	0.51	68	1.42	0.47	320
	**D90**	**20**	**3**	**36.1**	**6.6**	**1.93**	**26.7**	**0.41**	**72**	**1.47**	**nd**	**320**
	D180	-	-	-	-	-	-	-	-	-	-	-
19X	D0	24	3	33.7	7.8	2.32	27.5	0.42	28	0.99	0.28	320
	D30	22.4	3	35.2	7.6	2.36	28.4	0.45	26	0.93	0.5	320
	D60	23.9	2	40.2	7	2.44	25.5	0.54	24	0.99	0.2	160
	D90	23.7	4	41.9	7.5	2.36	25.1	0.46	27	1.01	nd	320
	**D180**	**22**	**5**	**35.2**	**7**	**2.25**	**22.5**	**0.47**	**21**	**0.76**	**nd**	**160**

*The bold values indicate the relapse timing*.

### Adverse Drug Reactions

In group X two dogs (4X, 8X) experienced diarrhea at D2, D3, and D4; a third dog (6X) had an episode of vomiting at D5. In group Y, one dog (16Y) presented vomiting and poorly formed stools for 1 day at beginning of therapy (D2) while a second dog (1Y) had a single episode of diarrhea at D2. For these five dogs, no supportive therapy was necessary. No long-term adverse effects were recorded. No statistical differences were found about the incidence of adverse events in the two groups.

## Discussion

Previous studies have shown that when used at a standard dosage, MIL improves the clinical score and allows the normalization of laboratory parameters during the time (5, 22, 24, 35). However, the complete clearance of parasites in blood, lymph nodes and BM could not be achieved in dogs despite a good clinical response (18, 19, 36), making these dogs susceptible to relapse (15, 35). The results of this pilot study highlighted a good safety and tolerability associated with good efficacy in terms of reduction of parasite load, clinicopathological improvement and reduction of relapses of the new proposed protocol compared to the conventional one.

Nevertheless, the high efficacy of Miltefosine-Allopurinol combination was confirmed (4, 37), with an improvement of the clinical score 2 months after the treatment in both groups, independently from the protocol. The percentage of reduction of clinical score from D0 to D180 was higher in group Y than in group X (71.6 and 61.7%, respectively) despite no significant differences were found.

The red blood cells values improved at D30 in both groups. The same timing for the improvement of these parameters was previously reported both with MA and with MIL at standard regimen (38). Nevertheless, it's possible to point out that hematological parameters showed a better positive trend throughout the study in group Y, compared to group X. Anemia is a common, but not constant sign in CanL. A high parasitic load in BM induces hematopoiesis abnormalities (39, 40) and BM disfunction. Thus, the better trend in hematological parameters observed in group Y could be attributable to the improved ability of the new dosing regimen of MIL to control the infection through the decrease of the BM parasitic load, as demonstrated by the qPCR results.

In this study, the A/G ratio progressively improved in both groups, but the improvement was faster in group Y, suggesting a MIL dosage-related effect on this parameter, as previously reported for MA (40). The A/G ratio is a marker of treatment response for CanL and the time it needs to reverse to normal is influenced by the initial pre-treatment value (41). In addition, the dog's response to therapy can be further monitored by recording the decrease of the IFAT titer, although this parameter does not constitute a good marker of the clinical improvement, mostly when considered alone (4). A correlation between the *Leishmania* antibody titer reduction, the clinical score trend and the decrease of the parasitic load has been reported (42–45). Accordingly, in our study the percentage of dogs that reached the cut-off or negative value at D180 was minimal (two dogs out of 34), confirming that positive IFAT values may persist in clinically recovered dogs (46, 47).

Bone marrow Real-Time PCR resulted negative in 50% of the dogs in group Y vs 14.3% of dogs in group X (Table 3). Moreover, the reduction of the parasitic load in group Y showed a more uniform trend reaching lower values close to be significant (*p*-value = 0.08) (Figures 2A,B). In this case, the lack of significance may have been influenced by the high variability of the parasitic load recorded in both groups at D0. With the aim of a comparison in two more homogeneous subgroups, we performed the *post-hoc* analysis on a limited number of dogs. Despite the limitation of this kind of analysis, it is interesting to point out the significant efficacy of the new therapeutic regimen on the reduction of parasitic load (*p*-value = 0.04).

The choice of the novel MIL dosage of 2.5 mg/kg daily was aimed to improve the efficacy of the treatment and it was based on the evidence that the same dosage (2.5 mg/kg/die) is used in human medicine for adults (48). Furthermore, in a recent study was demonstrated that a higher median daily dose of MIL, under allometric dosing regimen, provided an increased efficacy in children (49). On the other hands, dose determination studies on dogs under field condition showed that dogs treated with 4 mg/kg daily presented more frequent and severe side effects than dogs treated at 2 mg/kg (27).

It is known that MIL can induce some side effects on the gastrointestinal tract such as vomiting, abdominal pain and diarrhea; these clinical signs are usually mild, transient and self-limiting (19, 20, 25). In our study, the number of dogs that developed side effects was not significantly different between the two groups. The gastrointestinal manifestations were sporadic and limited during the first days of therapy. In both groups these signs have been attributed to the treatments after a clinical examination. The good tolerability of the higher dose of MIL for the treatment of CanL is probably due to a progressive drug adaptation of dogs, starting with a lower dosage than the usual. In our study dogs' food and feeding management was not changed, but we asked the owner to administer the drug always during feeding. It is already known that MIL adverse events are due to the direct effect of the drug on gastrointestinal tract following oral administration and not to a systemic effect (23, 26).

The authors hypothesize that it could exists a pre-systemic metabolism of MIL mediated by phospholipases at the level of the gastrointestinal epithelial cells with the release of degradation products (choline); these products are essential elements for the cellular membrane, thus they could be able to protect the gastrointestinal epithelial cells.

The MIL administration with food, better when fat food, is advised in human medicine (26), and in dogs adverse events are less frequent when MIL is administered with a complete meal rather than with a partial meal (25). For some authors, the possibility that a fatty diet could decrease the gastrointestinal effects derives from the detergent-like properties of MIL affecting the gastrointestinal lining (26). It is possible to argue that the measures adopted in our study (the reduced initial dosage and the drug administration during feeding) have limited the development of gastrointestinal adverse events in the novel protocol proposed. This aspect should be better pointed out in a large-scale study, because it could constitute an important issue in clinical practice, where many owners decide to stop therapy at onset of gastrointestinal signs, with a potential induction of drug resistance.

It has been documented that MIL has a low impact on renal function (23) and do not worse proteinuria (50). Serum levels of urea and creatinine persisted in normal range and UPC ratio did not worse throughout the study suggesting that MIL was safe also with the new protocol. In addition, UPC ratio showed a mild improvement in group Y at D60 suggesting a possible improvement of proteinuria in a shorter time.

The possibility and the percentage of relapses after treatment have been previously documented (35, 51). A double cycle of MIL has been proposed to reduce relapses with inconsistent results (24). In the present study the appearance of relapses only in dogs treated with the standard dose regimen, seems to be related with the better performance of the new protocol on parasitic load. If confirmed in a large-scale study, this result could be important to decrease the number of treatments with MIL with a consequent limitation of drug resistance, particularly desirable in countries where this drug is considered a first choice for the treatment of zoonotic visceral leishmaniasis in humans.

Despite being designed as a clinical pilot study, a limitation of this work is the lack of pharmacodynamic data on the novel miltefosine dosage. It was not possible to determine the MIL plasma concentration due to budget limitations.

Waiting for confirmation in large-scale studies the new dosage of MIL proposed in this pilot study showed a good potential for its applicability in practice, demonstrating a good safety and tolerability, with a similar or better trend of efficacy compared to the standard protocol, when referred to the reduction of parasitic load, clinical relapses and improvement of clinicopathological parameters.

## Data Availability Statement

The raw data supporting the conclusions of this article will be made available by the authors, without undue reservation.

## Ethics Statement

The animal study was reviewed and approved by Comitato etico per gli studi clinici e zootecnici veterinari del Dipartimento dell'Emergenza e dei Trapianti di Organo (D.E.T.O.) Prot.: DETO/163/2016; Approvazione: Consiglio del D.E.T.O. del 11.04.2016. Written informed consent was obtained from the owners for the participation of their animals in this study.

## Author Contributions

FI, PP, and MS: conceptualization, supervision, investigation, and writing. MC, BG, and MG: formal analysis. GTRR: resources. CN: conceptualization. VFM and GO: conceptualization, writing, and supervision. All authors contributed to the article and approved the submitted version.

## Conflict of Interest

The authors declare that the research was conducted in the absence of any commercial or financial relationships that could be construed as a potential conflict of interest.
