# Supplementary material for: Comparison of Two Dosing Regimens of Miltefosine, Both in Combination With Allopurinol, on Clinical and Parasitological Findings of Dogs With Leishmaniosis: A Pilot Study
Source: Front Vet Sci. 2020 Dec 14;7:577395. doi: 10.3389/fvets.2020.577395 (PMC7767967; doi:10.3389/fvets.2020.577395)
Supplement: Supplementary file 1 [file Data_Sheet_1.docx]

Group X: Miltefosine: 2 mg/kg BW, orally, once a day, for 28 consecutive days (Milteforan^®^Virbac, France). Group Y: Miltefosine: 1.2 mg/kg BW, orally, once a day, for 5 consecutive days (upper value) then 2.5 mg/kg BW, orally, once a day, for 25 consecutive days (Milteforan^®^Virbac, France)

| **GROUP X** | | | | | | **GROUP Y** | | | |
| --- | --- | --- | --- | --- | --- | --- | --- | --- | --- |
| **Case code** | | **Weight**  **(Kg)** | | **Daily**  **dose** | **Side effects** | **Case code** | **Weight**  **(Kg)** | **Daily**  **dose** | **Side effects** |
| **1X** | | 18 | | 36 |  | **1Y** | **17.5** | 21  43.75 | Diarrhea  at D2 |
| **2X** | | 18 | | 36 |  | **2Y** | **14.5** | 17.4  36.25 |  |
| **3X** | | 32 | | 64 |  | **3Y** | **21.5** | 25.8  53.75 |  |
| **4X** | | 50 | | 100 | Diarrhea at D2, D3 and D4 | **4Y** | **22** | 26.4  55 |  |
| **6X** | | 17 | | 34 | Vomit  at D5 | **5Y** | **28** | 33.6  70 |  |
| **7X** | | 22.5 | | 45 |  | **6Y** | **21** | 25.2  52.5 |  |
| **8X** | | 23 | | 46 | Diarrhea at D2, D3 and D4 | **7Y** | **38** | 45.6  95 |  |
| **9X** | | 15 | | 30 |  | **8Y** | **4** | 4.8  10 |  |
| **10X** | | 18 | | 36 |  | **9Y** | **15** | 18  37.5 |  |
| **11X** | | 10 | | 20 |  | **10Y** | **21** | 25.2  52.5 |  |
| **12X** | | 16 | | 32 |  | **11Y** | **8.3** | 9.96  20.75 |  |
| **13X** | | 38 | | 76 |  | **12Y** | **31** | 37.2  77.5 |  |
| **14X** | | 20 | | 40 |  | **13Y** | **16** | 19.2  40 |  |
| **15X** | | 25 | | 50 |  | **14Y** | **14** | 16.8  35 |  |
| **16X** | | 22 | | 44 |  | **15Y** | **26.5** | 31.8  66.25 |  |
| **17X** | | 16.8 | | 33.6 |  | **16Y** | **3** | 3.6  7.5 | Poorly formed stools at D2 |
| **18X** | | 32 | | 64 |  |  |  |  |  |
| **19X** | | 24 | | 48 |  |  |  |  |  |
|  |  | |  |  |  |  |  |  |  |
